# Physiological effects of awake prone position in acute hypoxemic respiratory failure

**DOI:** 10.1186/s13054-023-04600-9

**Published:** 2023-08-17

**Authors:** Domenico Luca Grieco, Luca Delle Cese, Luca S. Menga, Tommaso Rosà, Teresa Michi, Gianmarco Lombardi, Melania Cesarano, Valentina Giammatteo, Giuseppe Bello, Simone Carelli, Salvatore L. Cutuli, Claudio Sandroni, Gennaro De Pascale, Antonio Pesenti, Salvatore M. Maggiore, Massimo Antonelli

**Affiliations:** 1 Department of Emergency, Intensive Care Medicine and Anesthesia, Fondazione Policlinico Universitario A. Gemelli IRCCS, Rome, Italy; 2 Department of Anesthesiology and Intensive Care Medicine, Catholic University of the Sacred Heart, Fondazione ‘Policlinico Universitario A. Gemelli’ IRCCS, L.go F. Vito, 00168 Rome, Italy; 3 Department of Pathophysiology and Transplantation, University of Milan, Milan, Italy; 4 Department of Anesthesiology, Critical Care Medicine and Emergency, SS. Annunziata Hospital, Chieti, Italy; 5 University Department of Innovative Technologies in Medicine and Dentistry, Gabriele d’Annunzio University of Chieti-Pescara, Chieti, Italy

**Keywords:** Acute respiratory failure, Awake prone position, Inspiratory effort, High-flow nasal oxygen, Patient self-inflicted lung injury

## Abstract

**Background:**

The effects of awake prone position on the breathing pattern of hypoxemic patients need to be better understood. We conducted a crossover trial to assess the physiological effects of awake prone position in patients with acute hypoxemic respiratory failure.

**Methods:**

Fifteen patients with acute hypoxemic respiratory failure and PaO_2_/FiO_2_ < 200 mmHg underwent high-flow nasal oxygen for 1 h in supine position and 2 h in prone position, followed by a final 1-h supine phase. At the end of each study phase, the following parameters were measured: arterial blood gases, inspiratory effort (Δ*P*_ES_), transpulmonary driving pressure (Δ*P*_L_), respiratory rate and esophageal pressure simplified pressure–time product per minute (sPTP_ES_) by esophageal manometry, tidal volume (*V*_T_), end-expiratory lung impedance (EELI), lung compliance, airway resistance, time constant, dynamic strain (*V*_T_/EELI) and pendelluft extent through electrical impedance tomography.

**Results:**

Compared to supine position, prone position increased PaO_2_/FiO_2_ (median [Interquartile range] 104 mmHg [76–129] vs. 74 [69–93], *p* < 0.001), reduced respiratory rate (24 breaths/min [22–26] vs. 27 [26–30], *p* = 0.05) and increased Δ*P*_ES_ (12 cmH_2_O [11–13] vs. 9 [8–12], *p* = 0.04) with similar sPTP_ES_ (131 [75–154] cmH_2_O s min^−1^ vs. 105 [81–129], *p* > 0.99) and Δ*P*_L_ (9 [7–11] cmH_2_O vs. 8 [5–9], *p* = 0.17). Airway resistance and time constant were higher in prone vs. supine position (9 cmH_2_O s arbitrary units^−3^ [4–11] vs. 6 [4–9], *p* = 0.05; 0.53 s [0.32–61] vs. 0.40 [0.37–0.44], *p* = 0.03). Prone position increased EELI (3887 arbitrary units [3414–8547] vs. 1456 [959–2420], *p* = 0.002) and promoted *V*_T_ distribution towards dorsal lung regions without affecting *V*_T_ size and lung compliance: this generated lower dynamic strain (0.21 [0.16–0.24] vs. 0.38 [0.30–0.49], *p* = 0.004). The magnitude of pendelluft phenomenon was not different between study phases (55% [7–57] of *V*_T_ in prone vs. 31% [14–55] in supine position, *p* > 0.99).

**Conclusions:**

Prone position improves oxygenation, increases EELI and promotes *V*_T_ distribution towards dependent lung regions without affecting *V*_T_ size, Δ*P*_L_, lung compliance and pendelluft magnitude. Prone position reduces respiratory rate and increases Δ*P*_ES_ because of positional increases in airway resistance and prolonged expiratory time. Because high Δ*P*_ES_ is the main mechanistic determinant of self-inflicted lung injury, caution may be needed in using awake prone position in patients exhibiting intense Δ*P*_ES_.

*Clinical trail registeration*: The study was registered on clinicaltrials.gov (NCT03095300) on March 29, 2017.

**Supplementary Information:**

The online version contains supplementary material available at 10.1186/s13054-023-04600-9.

## Background

In intubated patients with moderate-to-severe acute respiratory distress syndrome, prone positioning reduces intrapulmonary shunt and generates lung recruitment, optimizes ventilation/perfusion matching, lowers alveolar dead space and reduces right ventricle afterload [1]. Through all these mechanisms, prone positioning improves gas exchange and attenuates ventilator-induced lung injury [2]. Robust evidence supports the systematic use of prone positioning to improve survival among intubated patients with moderate-to-severe acute respiratory distress syndrome [3, 4].

In recent years, prone position has been proposed in non-intubated patients with acute hypoxemic respiratory failure, with positive effects on arterial oxygenation [5–10]. A large randomized meta-trial and one observational study demonstrated that awake prone positioning in patients with hypoxemia due to COVID-19 reduces the need for endotracheal intubation and, possibly, mortality [11, 12]. However, other data did not confirm these findings and highlighted the possible risks related to intubation delays due to only transient oxygenation improvement produced by prone position [13, 14].

Despite plenty of clinical data obtained during the COVID-19 pandemic, few available studies address the physiological effects of prone position in spontaneously breathing humans with acute hypoxemic respiratory failure [15–17].

We conducted a sequential, crossover trial to comprehensively evaluate the effects of awake prone positioning on gas exchange, effort-to-breathe, lung volumes and inflation pattern in adult patients with moderate-to-severe acute hypoxemic respiratory failure undergoing high-flow nasal oxygen.

## Methods

This sequential crossover study was conducted in the intensive care unit of a university hospital in Italy between October 2018 and June 2020. The study was funded by an unrestricted research grant by the European Society of Intensive Care Medicine (ESICM-2017 Bernhard Dräger Award). The study was approved by institutional review board (ID 1506-ethics committee Fondazione Policlinico A. Gemelli IRCCS, Rome Italy) and was conducted in accordance with the declaration of Helsinki. All enrolled patients provided written informed consent to participating in the study and data analysis. The study protocol was registered on clinicaltrials.gov (NCT03095300) on March 29, 2017.

### Patients

Adult patients admitted to the intensive care unit due to acute hypoxemic respiratory failure were assessed for the enrolment. Acute hypoxemic respiratory failure was defined as an acute onset syndrome characterized by new or worsening impairment in oxygenation. Patients were considered eligible for inclusion if the following criteria were met: PaO_2_/FiO_2_ < 200, measured in the supine position while the patients was breathing heated and humidified high-flow oxygen through a non-rebreather face mask (60 L/min, temperature of the humidification chamber set at 37 °C, FiO_2_ set to achieve a SpO_2_ > 92% and < 98%—because of the high flows, nominal FiO_2_ was considered a reliable estimate of the actual one); PaCO_2_ < 45 mmHg; no of history of chronic respiratory failure or moderate-to-severe cardiac failure (New York Hear Association grade > II or left ventricular ejection fraction < 50%); body mass index < 30 kg/m^2^; absence of any contraindication to prone positioning (detailed in the Additional file 2: Supplementary material).

Exclusion criteria were: more than 48 h from the admission in the intensive care unit; acute exacerbation of asthma or chronic obstructive pulmonary disease; chest trauma; cardiogenic pulmonary edema; severe neutropenia (< 500 white blood cell count/mm^3^); hemodynamic instability (systolic blood pressure < 90 mmHg or mean arterial pressure < 65 mmHg) and/or lactic acidosis (serum lactate > 5 mmol/L) and/or clinically diagnosed shock; metabolic acidosis (pH < 7.30); chronic kidney failure requiring dialysis before intensive care unit admission; altered consciousness, defined by a Glasgow coma scale < 13; vomiting and/or upper gastrointestinal bleeding.

### Protocol

Patients received high-flow nasal oxygen for 1 h in the supine semirecumbent position. Gas flow was set at 60 L/min, the temperature of the humidification chamber (MR860 or ARIVO2, Fisher and Paykel healthcare) was set according to patient's comfort, FiO_2_ was titrated to maintain SpO_2_ > 92% and < 98%. After 1 h of high-flow treatment in the supine semirecumbent position, patients were placed in the prone position for 2 h and then placed again in the supine semirecumbent position to undergo a final hour of high-flow nasal oxygen.

For safety reasons, enteral feeding was interrupted 1 h before prone positioning and re-established after the study ended.

### Measurements

Patient’s demographics and main clinical characteristics were collected at study entry. During the study, each patient underwent standard monitoring including 5-lead electrocardiogram, invasive blood pressure and pulse oximetry. A polyfunctional nasogastric tube provided with an esophageal balloon (Nutrivent, Sidam, Italy) was placed and secured at a depth of 38–42 cm to measure esophageal pressure (*P*_ES_). The esophageal balloon was filled with 4 ml of air, which has been shown to be a non-stress volume providing reliability in a wide pressure range for the Nutrivent catheter [18]. To ensure intra-individual reproducibility, the esophageal balloon was deflated and, after checking adequate zeroing, re-inflated before all measurements. A pressure transducer measured *P*_ES_ (FluxMed v. 1802, MBMED, Buenos Aires, Argentina). An electrical impedance tomography (EIT) belt (LuMon, Sentec, Switzerland) with 16 electrodes was placed around the thorax between the fifth or sixth parasternal intercostal space and connected to a dedicated device to record electrical impedance signals. EIT data were acquired at a frame rate of 40 Hz. A detailed description of EIT signal processing procedure used in this study is provided elsewhere [19] and in Additional file 2: Supplementary material.

At the end of each step, the following data were collected: respiratory rate, SpO_2_, blood gases, heart rate, arterial pressure, dyspnea and discomfort as defined by a visual analogic scales (VAS) [19–21] (Additional file 1: E-Figures 1–2). EIT and *P*_ES_ were recorded for 15 min at the end of each study phase, once a stable breathing pattern was obtained.

*P*_ES_ and EIT signals were acquired in phase, amplified, low-pass filtered, digitalized at 40 Hz and stored in a personal computer (FluxMed v. 1802, MBMED, Buenos Aires, Argentina). All breaths from 15-min recordings were analyzed with MATLAB (Mathworks, Portola Valley, CA, USA). Results from all breaths in the 15-min recording were averaged for each study step.

### Endpoints

The primary objectives of this study were to assess the effects of prone position on arterial oxygenation (as defined by the PaO_2_/FiO_2_ ratio) and to establish the proportion of patients who underwent the procedure without displaying procedure-related serious adverse events, defined as any of the following: oxygen desaturations (SpO_2_ < 90%), hemodynamic instability (systolic arterial pressure < 80 mmHg or heart rate > 120 beats per minute), or displacement of central venous or arterial line.

Secondary endpoints of the study were the effects of prone position on:

- Breathing pattern: respiratory rate, inspiratory effort (the negative deflection of *P*_ES_ tracing during inspiration), *P*_ES_ simplified pressure time product (the chest wall recoil was neglected in all phases due to the impossibility of performing occlusions in non-intubated patients) [22, 23], VAS discomfort and dyspnea.
- Gas exchange: PaCO_2_ and corrected minute ventilation. Minute ventilation was expressed in arbitrary units and derived from the EIT signal: corrected minute ventilation was calculated as minute ventilation multiplied by the ratio of patient’s PaCO_2_ to 40 mmHg (with lower values indicating improved CO_2_ clearance, reduced CO_2_ production, or both) [24].
- Respiratory mechanics: The end-expiratory *P*_ES_, which reflects the superimposed pressure on dorsal lung zones [25], end-expiratory transpulmonary pressure (*P*_L_, calculated as airway pressure-*P*_ES_), quasi-static transpulmonary driving pressure (defined as the difference between end-inspiratory *P*_L_ and end-expiratory *P*_L_), airway resistance (Additional file 2: Supplementary material 3).
- Lung inflation pattern, measured with the EIT globally and regionally in the four regions of interests (ROI: ventral, mid-ventral, mid-dorsal, dorsal-Additional file 1: E-Figure 3—for the exact number of rows in each ROI refer to Additional file 2: Supplementary material 2). For this purpose, ROIs were defined from a standardized lung contour per patient and are specific for the impedance software used [19].

Analyzed EIT outcomes were: global and regional tidal volume, expressed in arbitrary units and calculated on a pixel-by-pixel basis; global and regional lung compliance, calculated as the ratio of tidal volume to quasi-static transpulmonary driving pressure; amount of pendelluft, expressed in terms of % of tidal volume (Additional file 2: Supplementary material 4) [19, 26–28]; end-expiratory lung impedance (EELI), expressed in arbitrary units and derived from the impedance signal and the lung strain definition [29–31], as described elsewhere [19]; global EELI and regional EELI distribution in the four ROIs; dynamic lung strain, computed as the ratio of tidal volume to the functional residual capacity: for this purpose, functional residual capacity was approximated to EELI; regional dynamic strain, computed as above, in the four ROIs; the amount of overstretched lung regions, defined as the percentage of lung pixels exhibiting dynamic strain greater than two [30]; inspiratory and expiratory times were assessed according to the time of zero flow (first derivative of the EIT signal), airway resistance calculated from the expiratory time constant as detailed in Additional file 2: Supplementary material and Additional file 1: E-Figure 4.

Respiratory mechanics and lung inflation pattern were analyzed according to a methodology described elsewhere [19] and in Additional file 2: Supplementary material. Consistently with a previous investigations, airway pressure during high-flow nasal oxygen was assumed to be constant and equal to 2.5 cmH_2_O [23, 24, 32]. For all calculations, beginning of inspiration and expiration was defined on the EIT vs. time tracing, when its first derivative became positive (end of expiration) and negative (end of inspiration).

### Sample size calculation

Given the physiological design of the study, we did not perform a formal sample size calculation. Consistently with previous investigations with similar design on the topic [19, 23, 24, 33, 34], we planned to enroll 15 patients, which is an adequate sample to draw significant conclusions on these specific endpoints.

### Statistical analysis

Categorical data are expressed as the event rate (%), while continuous data are expressed as the median [interquartile range]. Normality in the distribution of continuous variables was assessed with the Kolmogorov–Smirnov test.

Normally distributed quantitative variables in the three study steps were compared using ANOVA for repeated measures, with Bonferroni’s correction added for paired comparisons. Ordinal and non-normally distributed quantitative variables were analyzed using the Friedman test, with post hoc Dunn's test to adjust for multiple comparisons during pairwise testing of study phases. P-values, mean differences and confidence intervals for paired comparisons are displayed, and results with two-tail *p* ≤ 0.05 were considered statistically significant. For p-values greater than 0.01, two digits are provided and rounded to the closest second digit.

Correlations between continuous variables were assessed with Pearson’s correlation, and the r and p values are reported.

Statistical analysis was performed with SPSS 26.0, MATLAB R2021, and GraphPad Prism V 9.00.

## Results

The demographics and clinical characteristics of the enrolled patients are shown in Table 1. The median [interquartile range] PaO_2_/FiO_2_ at enrolment was 116 mmHg [97–127].

**Table 1:** Characteristic of patients at baseline

Age, years	66 [62–75]
Sex, female, *N* (%)	2 (13)
Height, cm	175 [170–178]
Body mass index, kg/m^2^	28 [24–30]
SAPS II^a^	31 [29–37]
SOFA at study inclusion^b^	2 [2–2]
COVID-19 as cause of respiratory failure, *N* (%)	8 (53)
Hematological malignancies, *N* (%)	5 (33)
*Duration of noninvasive respiratory support before enrolment, h*	
Noninvasive ventilation	0 [0–0]
Continuous positive airway pressure	0 [0–0]
High-flow nasal oxygen	0 [0–12]
Bilateral infiltrates at study inclusion^c^, *N* (%)	14 (93)
PaO_2_/FiO_2_ during face mask O_2_, mmHg	116 [97–127]
PaCO_2_ during face mask O_2_, mmHg	34 [27–36]
Glasgow Coma Scale score on inclusion	15 [15–15]
Need for endotracheal intubation, *N* (%)	4 (27)
Length of ICU stay, days	13 [5–19]
ICU mortality, *N* (%)	6 (40)

Data are expressed as medians [Interquartile range]. Unless specified otherwise

^a^SAPS II was calculated from 17 variables at enrollment. Information about previous health status. And information obtained at admission. Scores range from 0 to 163. With higher scores indicating more severe disease

^b^SOFA score was calculated from 6 variables at enrollment. Information about previous health status. And information obtained at admission. Scores range from 0 to 24. With higher scores indicating more severe disease

^c^All patients received chest X-ray the day of enrollment

**Fig. 1:**
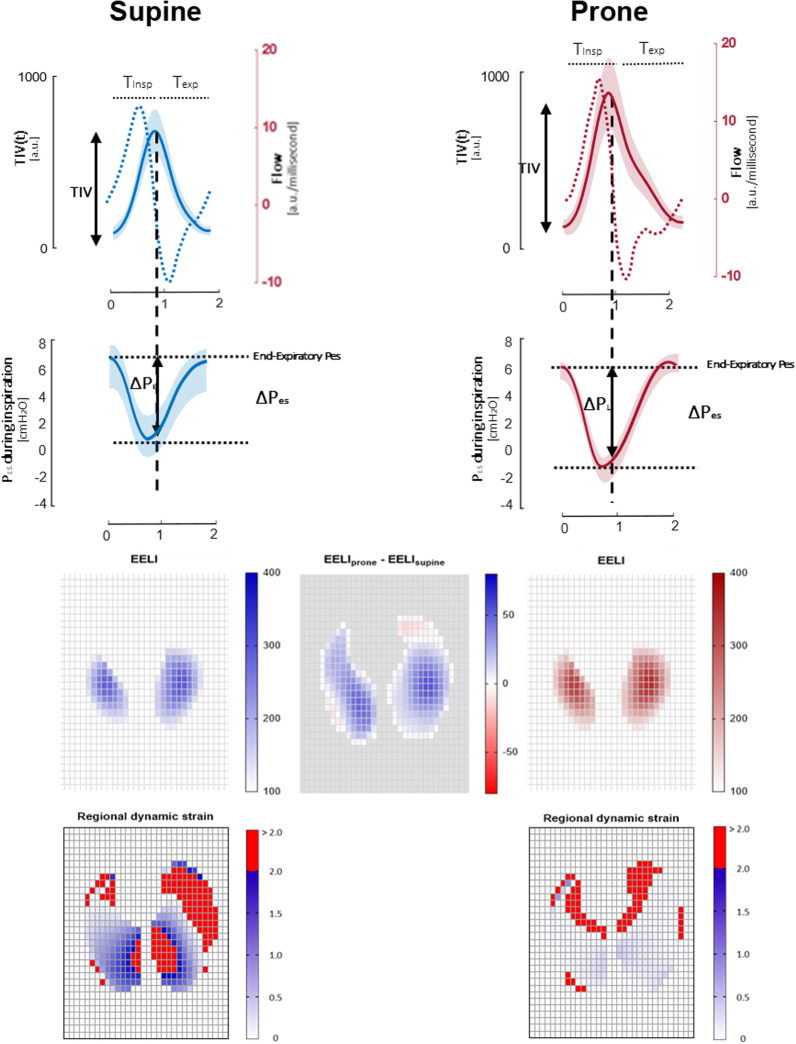
Tracings: comparisons between supine (in blue, left panel) and prone position (in red, right panel) for tidal volume (solid line), flow (dotted line), esophageal pressure. In the two top rows, average tidal impedance variation (TIV), flow and esophageal pressure are displayed. Average breaths from all patients were synchronized and interpolated. The resulting mean values (thick lines) and standard variation (shading) are displayed. Figures: end-expiratory lung volume increased and was dorsally shifted in the prone position. At the bottom, comparisons between the regional distribution of dynamic strain in the supine (left panel) and prone position (right panel). Pixel with a dynamic strain > 2 are displayed in red. These values represent the average values from the whole cohort

**Fig. 2:**
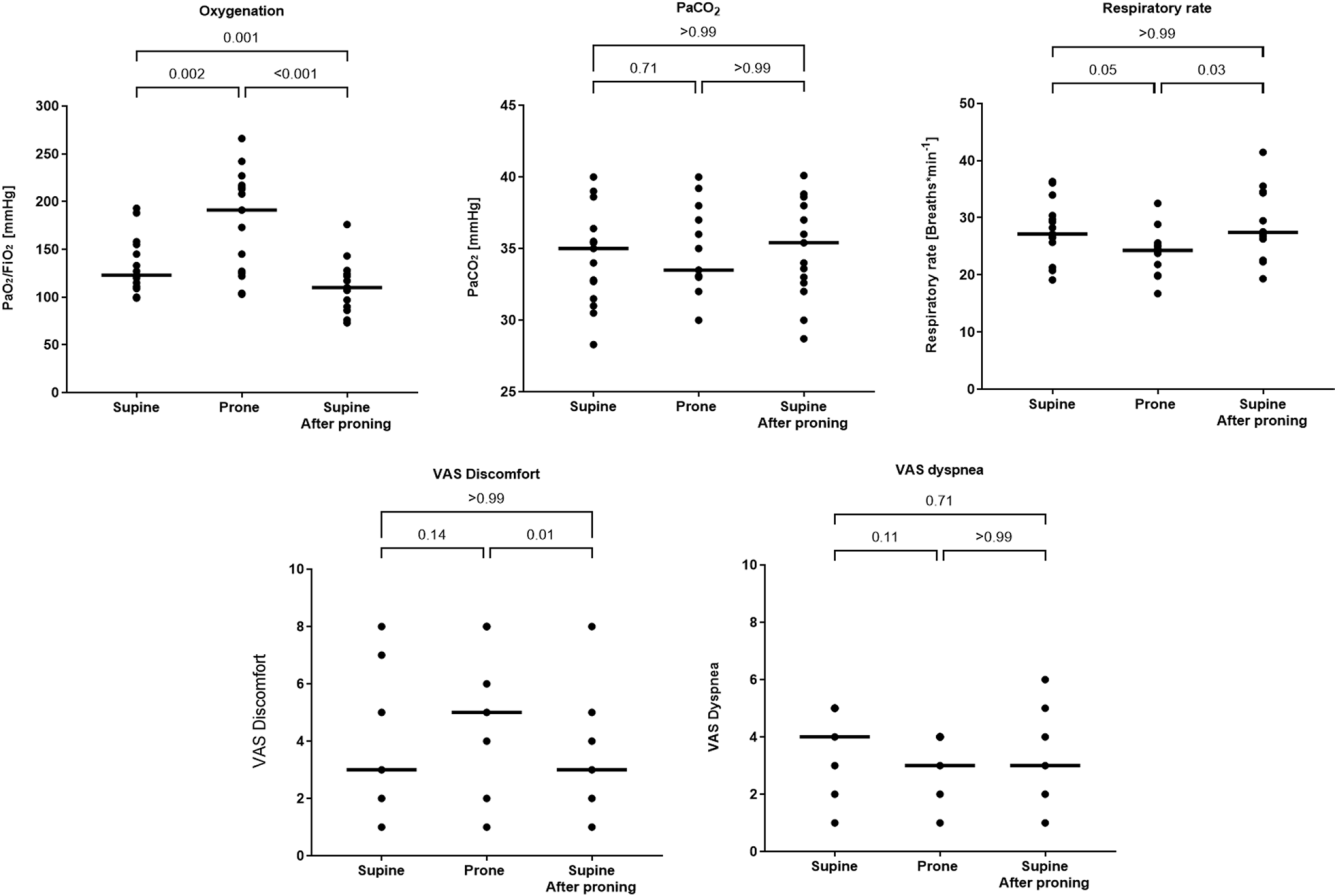
Individual patient values and medians of PaO_2_/FiO_2_, PaCO_2_, respiratory rate, and VAS-measured patient dyspnea and discomfort during the three phases of the study

**Fig. 3:**
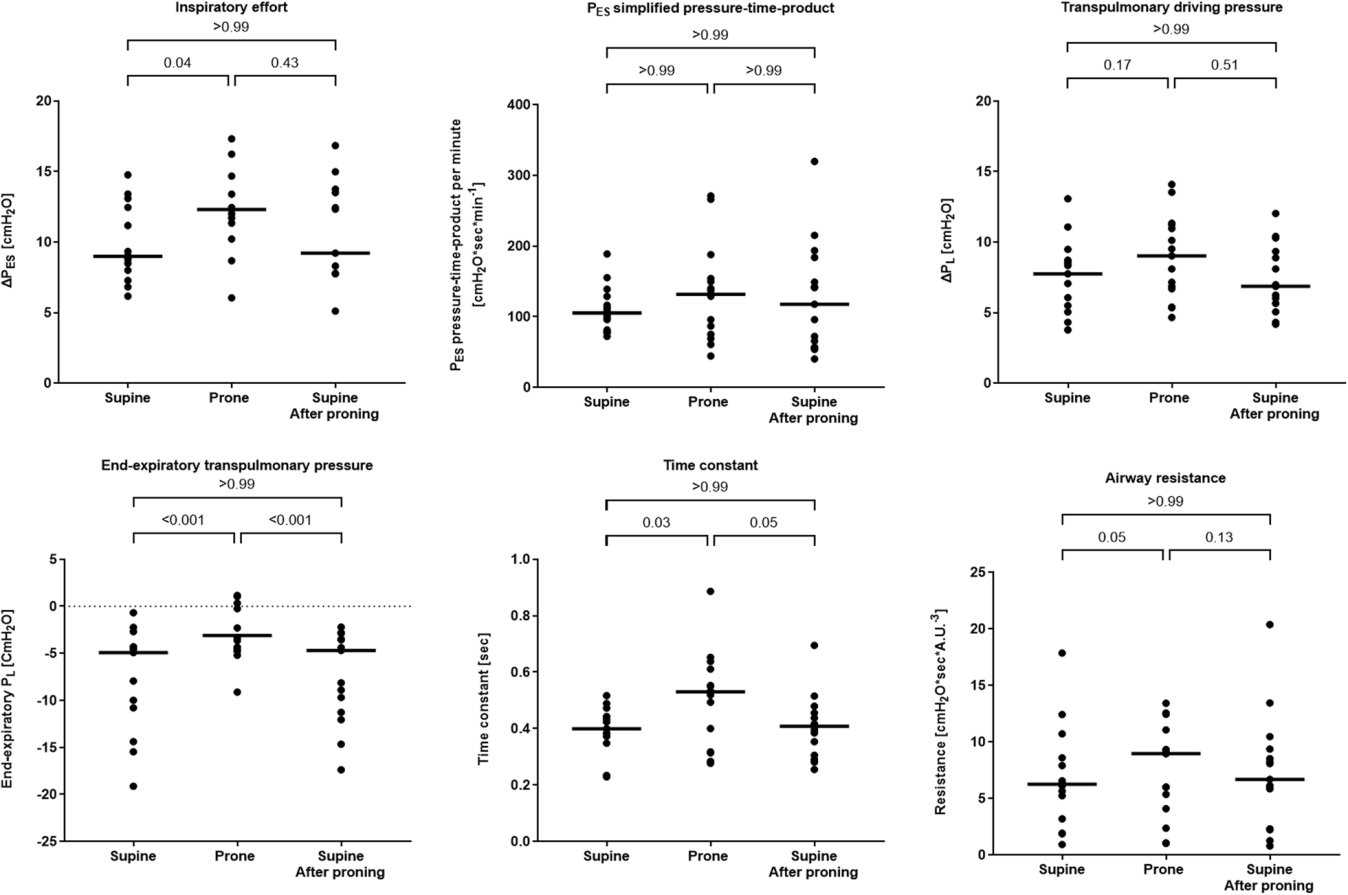
Individual patient values and medians of esophageal pressure inspiratory swings (Δ*P*_ES_), simplified pressure‒time product of the esophageal pressure per minute (PTP_ES_), quasi-static transpulmonary pressure (Δ*P*_L_), end-expiratory transpulmonary pressure, time constant and airway resistance during the three phases of the study

**Fig. 4:**
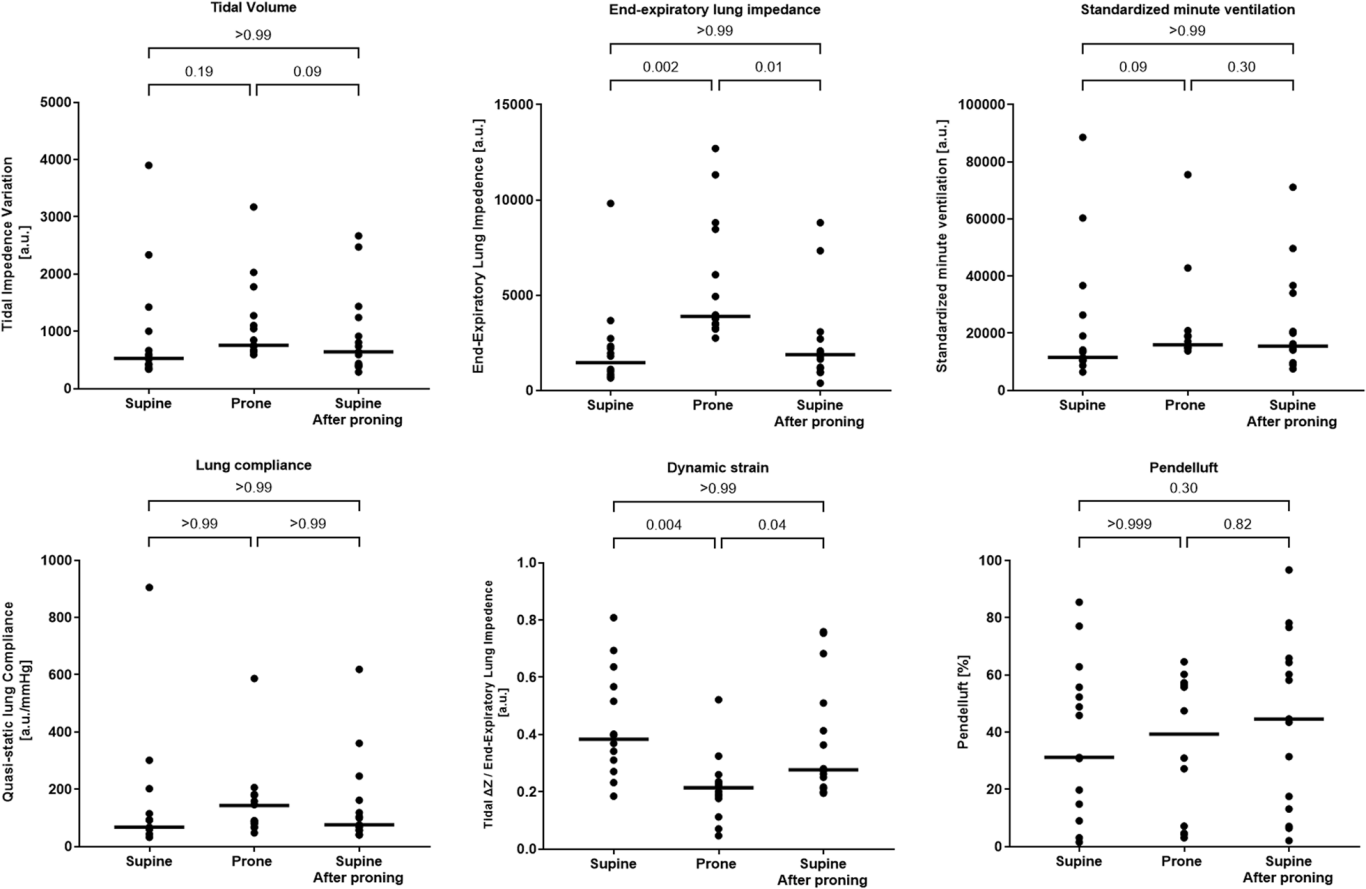
Individual patient values and medians of tidal impedance variation, end-expiratory lung impedance (EELI), standardized minute ventilation, lung compliance, dynamic strain and Pendelluft extent during the three phases of the study

Study results are displayed in Table 2 and Figs. 1, 2, 3, 4.

**Table 2:** Main results of the study

	Supine position	Prone position	Supine position after proning	*P* value^1^ Supine versus prone	*P* value^2^ Prone versus supine position after proning	*P* value^3^ Supine versus supine position after proning
*Gas exchange*						
FiO_2_	0.6 [0.6–0.6]	0.6 [0.6–0.6]	0.6 [0.6–0.6]	> 0.99	> 0.99	> 0.99
PaO_2_, mmHg	74 [69–93]	104 [76–129]	66 [54–73]	0.001	0.02	> 0.001
PaO_2_/FiO_2_, mmHg	123 [111–155]	191 [125–217]	110 [90–124]	0.002	< 0.001	0.001
SpO_2_, %	97 [96–99]	97 [96–99]	94 [90–95]	> 0.99	< 0.001	0.002
PaCO_2_, mmHg	35 [32–36]	34 [33–37]	35 [33–38]	0.71	> 0.99	> 0.99
*Self-assessed symptoms*						
Dyspnea, VAS	4 [2–4]	3 [1–4]	3 [1–4]	0.11	0.71	> 0.99
Self-assessed discomfort, VAS	3 [2–5]	5 [4–6]	3 [2–4]	0.14	0.01	> 0.99
*Respiratory mechanics*						
Esophageal pressure, end expiratory, cmH_2_O	7 [7–13]	6 [3–7]	7 [6–14]	< 0.001	> 0.99	< 0.001
Transpulmonary pressure, end expiratory^a^, cmH_2_O	− 5 [− 11 to − 4]	− 4 [− 5 to 0]	− 5 [− 11 to − 3.5]	< 0.001	< 0.001	> 0.99
Δ*P*_ES_, cmH_2_O	9 [8–12]	12 [11–13]	9 [8–14]	0.04	0.43	> 0.99
Δ*P*_L_, cmH_2_O	8 [5–9]	9 [7–11]	7 [6–9]	0.17	0.51	> 0.99
Inspiratory time, s	1.05 [0.90–1.32]	0.95 [0.90–1.20]	1.00 [0.77–1.25]	> 0.99	> 0.99	> 0.99
Expiratory time, s	1.23 [0.96–1.41]	1.44 [1.38–1.49]	1.31[1.15–1.57]	0.05	0.43	> 0.99
Respiratory rate, breaths per minute	27 [26–30]	24 [22–26]	27 [23–34]	0.05	0.03	> 0.99
Simplified *P*_ES_ pressure–time-product per minute, cmH_2_O s min^−1^	105 [81–129]	131 [75–154]	117 [57–184]	> 0.99	> 0.99	> 0.99
Time constant (s)	0.40 [0.37–0.44]	0.53 [0.32–0.61]	0.41 [0.31–0.46]	0.03	0.05	> 0.99
Resistance (cmH_2_O s A.U.^−3^)	6 [3–9]	9 [4–11]	7 [2–10]	0.05	0.13	> 0.99
*EIT-derived indices*						
Standardized minute ventilation (arbitrary unites/minute)	11524 [8959–26376]	15927 [14673–20894]	15522 [9062–34072]	0.09	> 0.99	0.30
*TidalΔZ, arbitrary units*	528 [418–1004]	756[669–1275]	643[401–1244]	0.08	0.08	> 0.99
Ventral ROI	27 [12–65]	61 [16–80]	42 [29–53]	0.01	> 0.99	0.13
Mid-ventral ROI	78 [50–233]	102 [55–397]	171 [32–303]	> 0.99	> 0.99	> 0.99
Mid-dorsal ROI	200 [164–566]	279 [255–580]	253 [145–499]	0.09	> 0.99	0.09
Dorsal ROI	54 [37–184]	122 [87–225]	87 [35–213]	0.05	> 0.99	0.43
Pendelluft, % of TidalΔZ	31 [14–55]	55 [7–57]	44 [13–66]	> 0.99	0.30	0.82
*Lung Compliance, Arbitrary units/cmH*_*2*_*O*	67 [60–115]	89 [79–179]	76 [57–162]	> 0.99	> 0.99	> 0.99
Ventral ROI	2 [2–9]	7 [2–10]	5 [4–7]	0.13	0.60	> 0.99
Mid-ventral ROI	12 [7–21]	10 [7–35]	20 [7–43]	> 0.99	> 0.99	> 0.99
Mid-dorsal ROI	28 [20–67]	38 [26–81]	23 [17–80]	> 0.99	> 0.99	> 0.99
Dorsal ROI	7 [5–21]	16 [10–22]	10 [7–26]	0.13	0.60	> 0.99
*EELI, arbitrary units*	1456 [959–2420]	3887 [3414–8547]	1879 [1114–4143]	0.002	0.01	> 0.99
Ventral ROI	73 [49–105]	265 [111–390]	106 [43–198]	< 0.001	0.02	> 0.99
Mid-ventral ROI	492 [293–786]	1195 [1061–2372]	573 [371–1187]	0.002	0.01	> 0.99
Mid-dorsal ROI	736 [440–1288]	1794 [1656–4325]	849 [550–2029]	< 0.001	0.004	> 0.99
Dorsal ROI	194 [129–340]	615 [494–1015]	299 [131–583]	0.002	0.01	> 0.99
*Dynamic strain*	0.38 [0.30–0.59]	0.21 [0.16–0.24]	0.27 [0.22–0.55]	0.004	0.04	> 0.99
Ventral ROI	0.35 [0.23–0.58]	0.23 [0.12–0.29]	0.34 [0.28–0.77]	0.04	0.02	> 0.99
Mid-ventral ROI	0.20 [0.12–0.32]	0.07 [0.04—0.15]	0.19 [0.08—0.43]	0.01	0.14	> 0.99
Mid-dorsal ROI	0.34 [0.21–0.50]	0.16 [0.10–0.24]	0.23 [0.19–0.31]	< 0.001	0.11	0.39
Dorsal ROI	0.32 [0.24–0.42]	0.17 [0.15–0.25]	0.31 [0.15–0.54]	0.27	> 0.99	0.77
*Hemodynamics*						
Heart rate, beats per minute	72 [62–80]	74 [65–78]	75 [61–84]	0.72	0.13	0.55
Arterial pressure, mmHg						
Systolic	138 [120–151]	136 [122–155]	132 [112–149]	0.44	0.45	0.86
Diastolic	72 [58–90]	70 [58–81]	66 [55–87]	0.32	0.45	0.12

Data are reported as medians [interquartile ranges]

All paired comparisons were adjusted with Bonferroni’s or Dunn’s correction. As appropriate. *P* values ≤ 0.05 are considered statistically significant

^a^The airway pressure during HFNO was not measured but assumed to be constant at 2.5 cmH_2_O

### Gas exchange and subjective symptoms

None of the fifteen enrolled patients experienced any serious adverse events. Thirteen out of 15 patients showed increased blood oxygenation after 2 h of prone positioning (mean difference 45 mmHg [95% CI 23–68], *p* = 0.002). However, upon re-supination a decrease in oxygenation was observed compared with prone positioning (mean difference − 67 mmHg [95% CI 94–41], *p* < 0.001) and supine position before pronation (mean difference − 22 mmHg [95% CI − 12 to − 32], *p* = 0.001) (Fig. 2).

PaCO_2_ did not change during any of the positional changes (*p* = 0.47) (Fig. 2).

There was no change in perceived dyspnea among the three study phases, but patients exhibited less tolerability for the prone position when resupinated (*p* = 0.01) (Fig. 2).

### Effort to breath and respiratory mechanics

Respiratory rate decreased during prone positioning (mean difference − 2 breaths per minute [95% CI − 6 to − 1], *p* = 0.05), a benefit not maintained after re-supination (Fig. 2).

Compared to supine position, prone position increased inspiratory effort (Δ*P*_ES_ mean difference 2 cmH_2_O [95% CI 1–4], *p* = 0.04) (Fig. 3). During prone position, the increase in Δ*P*_ES_ was not accompanied by changes in Δ*P*_L_ (*p* = 0.63).

Prone position was associated with changes in respiratory system resistive properties, with an increased time constant compared to both supine phases (*p* = 0.03, *p* = 0.05). This was caused by increased airway resistance (*p* = 0.05) with prolonged expiratory time (*p* = 0.05) during prone position.

Prone-induced changes in airway resistance were related to the change in Δ*P*_ES_ (r = 0.53, *p* = 0.04) (Additional file 1: E-Figure 5).

Simplified minute PTP_ES_ did not differ between any of the study phases (*p* > 0.05 for all comparisons) (Fig. 3).

In prone position, end-expiratory esophageal pressure was lower (*p* < 0.001), and end-expiratory transpulmonary pressure was higher (*p* = 0.03) than during supine position (Fig. 3).

### Tidal volume

**Fig. 5:**
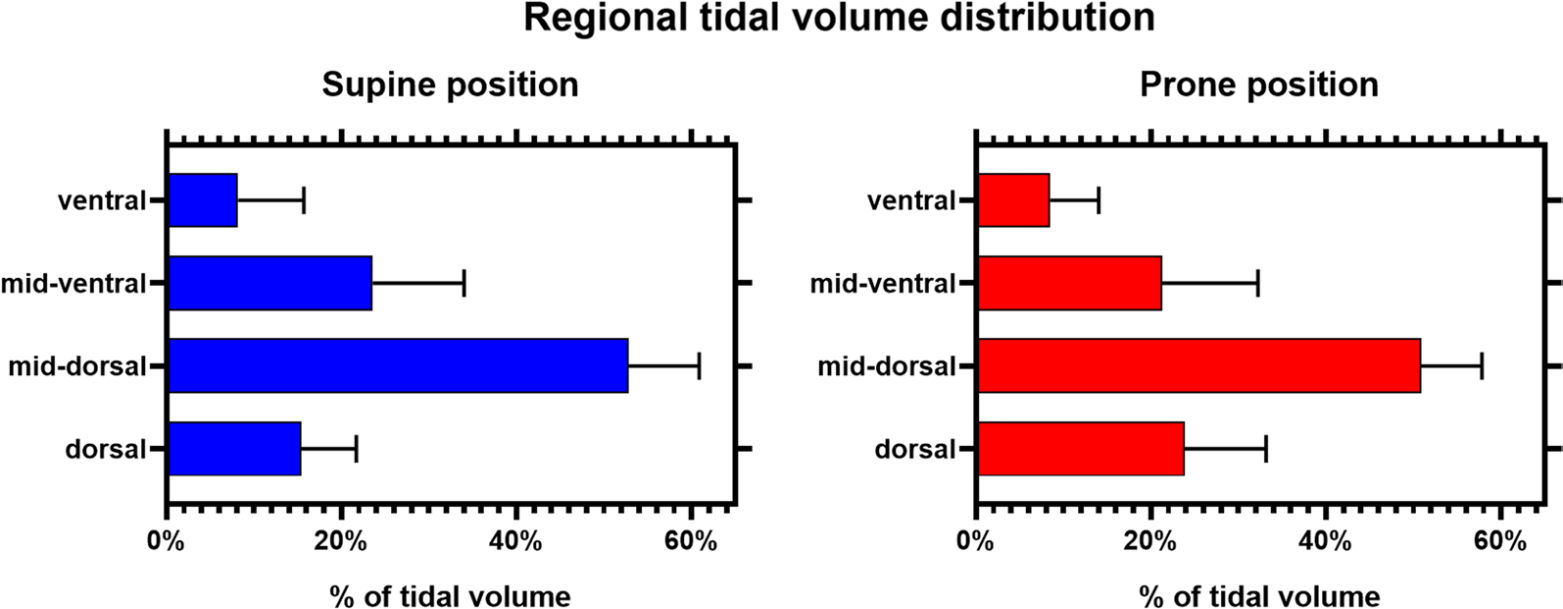
Tidal volume distribution (expressed in % of global tidal volume) in supine and prone position. Results are expressed as means (standard deviation). Prone position promoted tidal volume distribution towards dorsal, dependent lung regions

Compared to supine position, prone position did not yield changes in tidal volume (all *p* > 0.05) (Fig. 4). Prone position altered tidal volume distribution, resulting in a significant increase in ventilation of the ventral ROI (mean difference 20% [95% CI 3–37], *p* = 0.01) and dorso-dorsal ROI (mean difference 35% [95% CI − 8 to 78], *p* = 0.05) compared to supine position (Fig. 5**)**.

Given the unchanged Δ*P*_L_ and tidal volume, no changes in lung global and regional compliance were detected (*p* > 0.05 for all comparisons).

Pendelluft was common in our cohort (31% [14–55] of tidal volume during supine position, 55% [7–57] during prone position, 44% [13–66] after re-supination), without significant differences between treatments (*p* > 0.05 for all comparisons).

### End-expiratory lung impedance

Prone position increased EELI compared to supine phases before and after the intervention (mean % increase 279% [95%CI: 133 to 330], *p* = 0.002 and 106% [95%CI: 22 to 448] arbitrary units, *p* = 0.01, respectively) (Fig. 4, Table 2).

The increase in EELI occurred throughout all lung regions, but was prominent in dorsal ROIs.

The increase in EELI led to a reduction in the dynamic strain during prone position, compared to both supine positions before and after pronation (mean difference − 0.22 [95% CI − 0.33 to − 0.11], *p* = 0.004; − 0.17 [95% CI − 0.30 to − 0.04], *p* = 0.04, respectively). No difference was observed in dynamic strain between the two supine phases (*p* > 0.99). Dynamic strain was predominantly ventral in supine position and predominantly dorsal in prone position (Table 2).

## Discussion

The results of this sequential study on the physiological effects of awake prone positioning in patients with moderate-to-severe hypoxemic respiratory failure undergoing high-flow nasal oxygen can be summarized as follows:

- Awake prone positioning improves arterial oxygenation without serious periprocedural adverse events. However, the benefit on oxygenation is transient and, after supination, oxygenation may significantly worsen, likely reflecting patient’s deterioration.
- Awake prone positioning reduces respiratory rate but increases Δ*P*_ES_, with no effects on Δ*P*_L_, *V*_T_, quasi-static lung compliance, simplified minute PTP_ES_, PaCO_2_ and dyspnea. These effects are mediated by increased airway resistance to flow throughout all the respiratory cycle, with higher expiratory time constant and prolonged expiration.
- Awake prone positioning increases EELI. This occurs due to recruitment of dorsal lung regions, enhanced expiratory pressure produced by higher resistance to expiratory flow and higher end-expiratory transpulmonary pressure.
- In prone position, the increase in EELI combined to a shift of the *V*_T_ towards dorsal lung regions causes a reduction in global and regional lung strain, reflecting more homogenous lung inflation.
- Prone positioning does not affect the magnitude of the pendelluft phenomenon.

In intubated patients with moderate-to-severe ARDS, prone position improves oxygenation, limits ventilator-induced lung injury and decreases mortality [1, 3, 4]. During the COVID-19 pandemic, awake prone position has been extensively applied, with favorable effects on arterial oxygenation [5]. Subsequent randomized studies confirmed the beneficial effects on oxygenation, but showed conflicting results on the capability of awake prone position to reduce the rate of endotracheal intubation and improve patient-centered outcomes [12, 14].

Over the past decade, the management of patients with hypoxemic respiratory failure has changed significantly. High-flow nasal oxygen has emerged as a means of avoiding endotracheal intubation and minimizing the adverse effects of sedation and invasive mechanical ventilation [35, 36]. However, intubation after noninvasive support is still required in many cases, and failure of noninvasive support results in increased mortality [37–39]. A better understanding of the physiology of spontaneous breathing has given rise to a new clinical challenge: the careful balance between using noninvasive devices to avoid intubation vs. the risk of exposure to delayed intubation combined with harmful spontaneous breathing leading to patient self-inflicted lung injury (SILI). SILI arises from increased Δ*P*_ES_ and lung inhomogeneity, which cause uneven lung inflation and local overstretch, especially in the dependent lung [40]. This partially differs from ventilator-induced lung injury caused by controlled mechanical ventilation, which mostly occurs in the non-dependent lung exposed to mechanical stress and strain imposed in the absence of spontaneous breathing [41].

The major SILI determinant is the intensity of Δ*P*_ES_ [42]: accordingly, Δ*P*_ES_ and its changes as a response to treatment represent the most relevant determinants of the subsequent need for intubation during noninvasive support [43]. This indicates that any noninvasive intervention should be interpreted addressing its effect both on oxygenation and Δ*P*_ES_, with Δ*P*_ES_ reflecting the risk of SILI [44].

Several studies showed that awake prone position improves oxygenation, but few studies elucidated the effects of awake prone positioning on Δ*P*_ES._ In an animal lung injury model, prone position was shown to reduce Δ*P*_ES_ and minimize SILI [45]. However, subsequent studies in non-intubated humans with acute hypoxemic respiratory failure did not confirm these findings, and showed that prone position mainly reduces respiratory rate, with unchanged, or even increased, Δ*P*_ES_ [16, 17]. This discrepancy between animals and humans may be explained by the specific features of experimental lung injury and the animals studied, whose posture is naturally prone.

Our study confirms that prone position can improve oxygenation without serious adverse events related to the procedure. This is attributable to the observed increase in EELI, caused by the positional change and, likely, to enhanced positive expiratory pressure produced by increased airway resistance to expiratory flow and higher transpulmonary end-expiratory pressure [46]. Prone-induced increases in functional residual capacity have been documented in non-intubated humans since 1960s [47] and are consistent with the mechanism of action of prone position in intubated patients with acute respiratory distress syndrome [3]. In patients with acute hypoxemic respiratory failure, improvement in oxygenation may help avoid endotracheal intubation, since hypoxemia is a relevant cause of treatment failure during noninvasive support [48]. However, in our study, oxygenation significantly worsened (even compared to study start) when the patient was re-placed in supine position. Other investigators have reported this phenomenon [13, 14], but the exact mechanism behind this is unknown.

In our cohort, prone positioning reduced respiratory rate but increased Δ*P*_ES_. The reduction in respiratory rate is a well-documented effect of prone position in spontaneously breathing patients [15–17]. Higher Δ*P*_ES_ in the prone position has been previously observed [17]. Our results indicate that the increase in Δ*P*_ES_ in prone position is induced by increased airway resistance to flow, with unchanged *V*_T_, lung compliance and quasi-static transpulmonary driving pressure. Δ*P*_ES_ includes the resistive and elastic workload per breath: in our study, prone position increased the resistive workload, with unchanged elastic workload. Increased expiratory resistance (i.e., limited expiratory flow) in prone vs. sitting position has been shown in healthy individuals [49, 50] and may occur due to small airway closure and gravitational changes in lower airways [51]. Moreover, prone position necessitates breathing with the head in a laterally rotated position, which causes dealignment of the cranio-pharyngeal axis: this can increase resistance due to geometrical changes in the shape of upper airways and a heightened risk of turbulent flow [52, 53]. All these mechanisms explain the prone-induced increase in Δ*P*_ES_ (higher respiratory resistance) and reduction in respiratory rate (longer expiratory time constant due to higher expiratory resistance).

In our study, we neglected the amount of Δ*P*_ES_ needed to overcome chest wall recoil pressure, as we were unable to perform occlusions. This is particularly relevant during the prone position due to an increase in chest wall elastance [54]. As a result, we may have underestimated the Δ*P*_ES_ during this phase more than in supine position, and the actual increase in Δ*P*_ES_ during prone position is likely higher than we were able to demonstrate.

In our study, prone position reduced global and regional dynamic strain, which represents the most relevant determinant of ventilator-induced lung injury during controlled ventilation in patients with acute respiratory distress syndrome. This may contribute to the beneficial effects of prone position observed in spontaneously breathing subjects in clinical and observational studies [12, 45, 55].

In our study, prone position did not affect the pendelluft phenomenon, which is one of the mechanisms of SILI [40, 42]. The primary determinant of the pendelluft phenomenon is Δ*P*_ES_ [19, 40, 42], that was not reduced, and even increased, with prone position.

Our results have relevant clinical implications:

- Prone-induced improvement in oxygenation may help avoid endotracheal intubation, since hypoxemia is a relevant cause of treatment failure during noninvasive support [48]. However, oxygenation significantly worsened (even compared to study start) when the patient is placed again in supine position. This may yield intubation delays, with possible detrimental effects on clinical outcome. This may also explain the dose–response relationship between the intervention and clinical outcome observed in a clinical trial [12], where a higher duration of prone sessions led to reduced risk of subsequent endotracheal intubation.
- Prone position homogenizes lung inflation, reduces global and regional dynamic strain and respiratory rate, does not affect the amplitude of the pendelluft phenomenon, *V*_T_, lung compliance and quasi-static driving pressure, and increases Δ*P*_ES_. Although dynamic strain is the main determinant of ventilator-induced lung injury, Δ*P*_ES_ and pendelluft are major determinants of SILI [12, 56]. Prone position appears to mitigate the risk of lung injury due to dynamic strain, but not the injury due to Δ*P*_ES_ and pendelluft. Also, high Δ*P*_ES_ may yield diaphragm injury and muscle exhaustion, which may cause treatment failure and are associated with worse long-term clinical outcome [57, 58]. Interestingly, in a previous study, the entity of Δ*P*_ES_-increase due to prone position was associated with the subsequent need for intubation [17]. Although the absolute increase in Δ*P*_ES_ was limited in our cohort, our results may indicate that awake prone positioning may not be indicated for all patients with acute hypoxemic respiratory failure, but only for those exhibiting low-to-normal Δ*P*_ES_. Those with high Δ*P*_ES_ are rather likely to benefit from an approach specifically aimed at reducing the inspiratory effort: this may explain why noninvasive ventilation (that best reduces Δ*P*_ES_) may result in reduced rate of intubation vs. high-flow nasal oxygen combined with prone position among patients with high Δ*P*_ES_ [19, 59–61]. Discrepancy between the huge clinical benefit by prone positioning in intubated patients vs. the milder observed in non-intubated patients may be related to the inability of the intervention to modulate Δ*P*_ES_.

Our study has limitations. First, we assessed the effects of prone positioning after 2 h, while it has been shown that the most clinical benefit by prone positioning is observed in patients who remain prone for longer periods [12]; however, our design is consistent with that of studies addressing the physiological effects of prone positions in intubated patients [54]. Second, because of the impossibility to perform occlusions, we neglected the respiratory workload related to chest-wall recoil pressure: since prone position increases chest wall elastance, this should not alter, and could even strengthen, our results on Δ*P*_ES_. Third, we assumed that inspiratory and expiratory resistance were equal, while they can be different: assessing resistance in non-intubated patients is complex, and we deem this may be an acceptable approximation. Fourth, prone-induced increases in airway resistance may have affected end-expiratory pressure, which was assumed to be constant in our study: although this might have slightly changes our dynamic strain calculations, we believe this does not affect the overall meaning of the investigation. Fifth, our physiological measurements were obtained with electrical impedance tomography, which provides data in arbitrary units rather than in volume/flow; however, the electrical impedance tomography signal is strictly linked with changes in lung aeration [62], and the crossover design of our study allows to detect the changes induced by the intervention, independently from the absolute values of flow and volume in the study phases. Sixth, absolute values of EELI rely on the physiological relationship between stress and strain; therefore, absolute EELI and dynamic strain values should be interpreted cautiously. Finally, we did not measure gastric pressure, making impossible to establish whether expiratory muscles recruitment induced by body position may have contributed to the observed results.

## Conclusions

In patients undergoing high-flow nasal oxygen and exhibiting moderate-to-severe hypoxemia due to acute respiratory failure, prone position improves oxygenation by enhancing recruitment of dorsal lung regions, homogenizes ventilation distribution and reduces respiratory rate. Prone position does not affect *V*_T_, the amount of pendelluft, transpulmonary driving pressure and lung compliance, but increases Δ*P*_ES_ because of higher airway resistance with longer expiratory time constant. Because Δ*P*_ES_ and pendelluft are the main mechanistic determinants of SILI, caution may be needed in the use of prone position in patients exhibiting high Δ*P*_ES_.

### Supplementary Information

**Additional file 1**. **E – Figure 1:** VAS dyspnea scale.**E – Figure 2:** VAS discomfort scale. **E – Figure 3:** Graphic representation of the 4 ROIs and lung contour. In the picture there is a representation of the 32 x 32 matrix provided by the FluxMed® device, after filtering for hearth artifacts and isolating the lungs. In order, from ventral to dorsal: ventral ROI, mid-ventral ROI, mid-dorsal ROI, dorsal ROI. In the analysis, as per manufacturer instructions, the EIT value of each pixel was adjusted to its position in the lung image, with the most peripherical and ventral pixels having the lowest value. **E – Figure 4:** Graphic representation of the exponential fitted curve in a representative single breath cycle of supine position. The thin gray line represents the raw data with artifacts on which a moving-average smoothing is applied (blue line). The red curve is the negative exponential fit applied (eq. 1). The starting point was chosen as the 75% of the maximum value of the TIV and the end point as the minimum value of the TIV(t).**E – Figure 5:** Relationship between the change in inspiratory effort and the change in resistances across study phases. In the top two panels, a significant linear relationship is depicted between the increase in respiratory system resistances and inspiratory effort when comparing the supine phases (before and after re-supination) to the prone phase. In the bottom two panels, the same relationship is shown between the change in respiratory system resistances and ΔPL_2_ across the study phases; however, in this case, the relationship is not significant.

**Additional file 2**. Supplementary material.

## Data Availability

The datasets used and/or analyzed during the current study are available from the corresponding author on reasonable request.

## Abbreviations

ARDS: Acute respiratory distress syndrome
PEEP: Positive end-expiratory pressure
Δ*P*_L_: Transpulmonary driving pressure
Δ*P*_ES_: Inspiratory effort
sPTP_ES_: Simplified esophageal pressure pressure–time product
*V*_T_: Tidal volume
EELI: End-expiratory lung impedance
ROI: Region of interest
