# Supplementary figures and images for: Physiological effects of awake prone position in acute hypoxemic respiratory failure
Source: Crit Care. 2023 Aug 17;27:315. doi: 10.1186/s13054-023-04600-9 (PMC10433569; doi:10.1186/s13054-023-04600-9)

## Slide 1
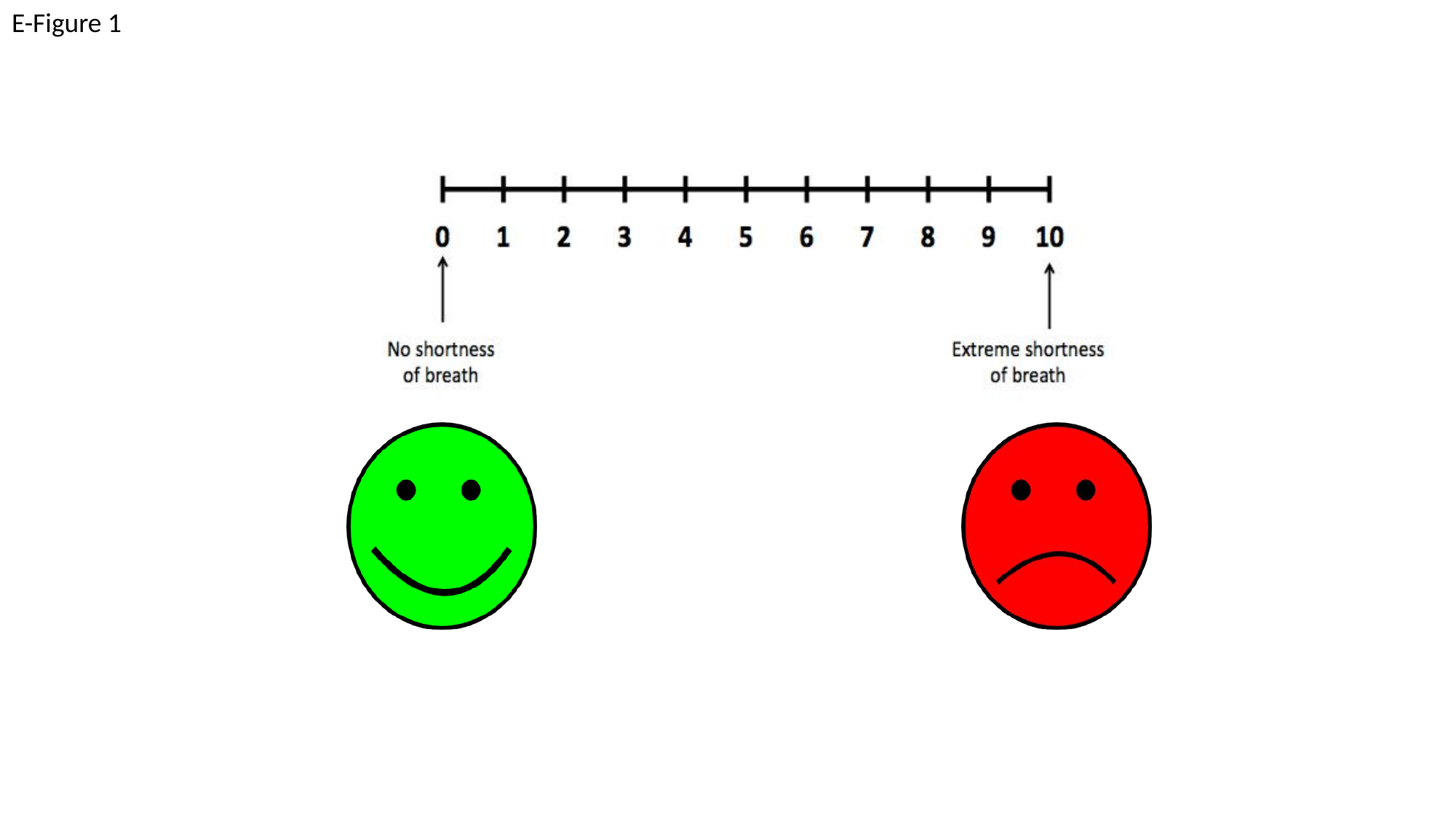

E-Figure 1

## Slide 2
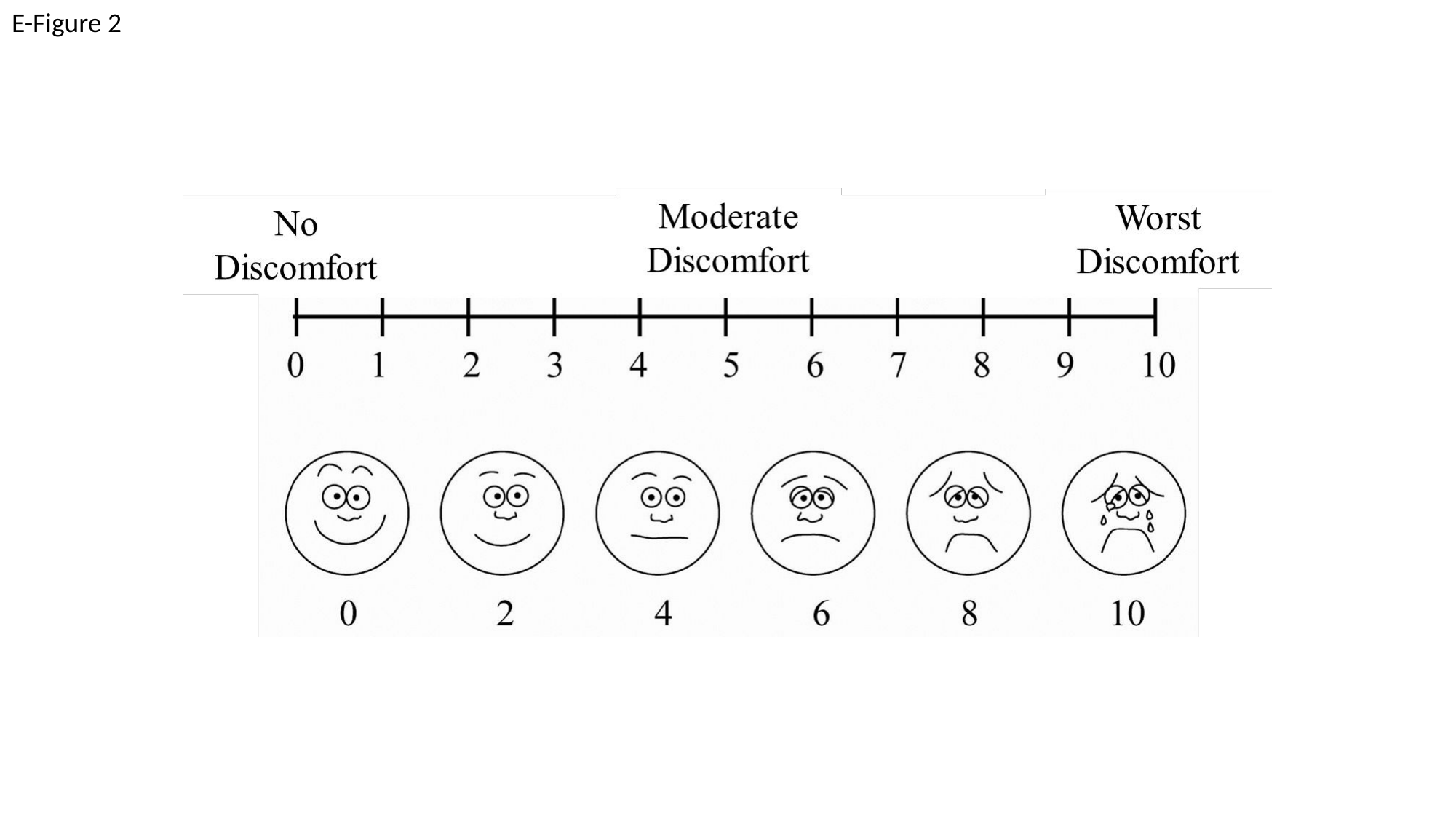

E-Figure 2

## Slide 3
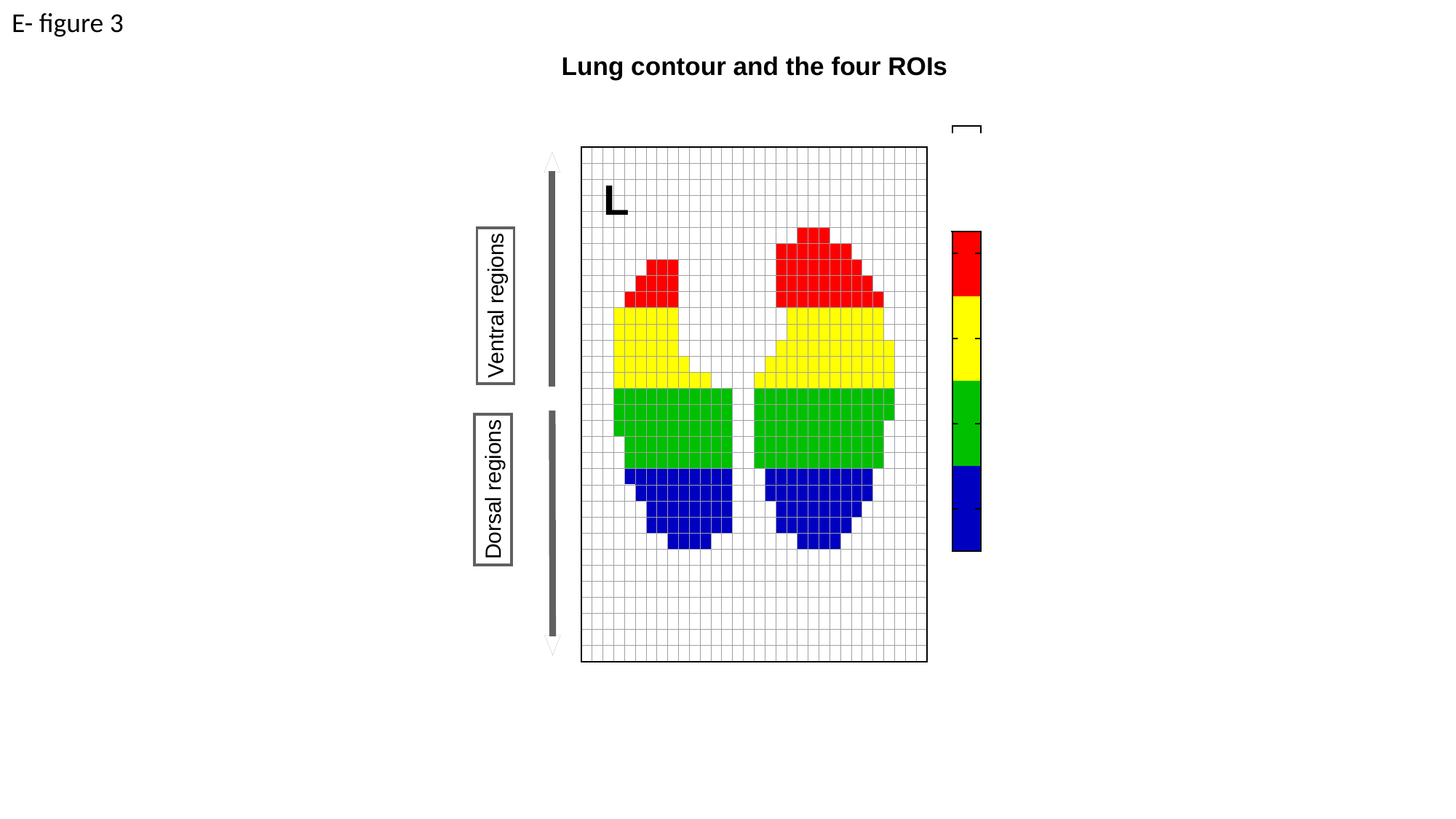

E- figure 3
L

## Slide 4
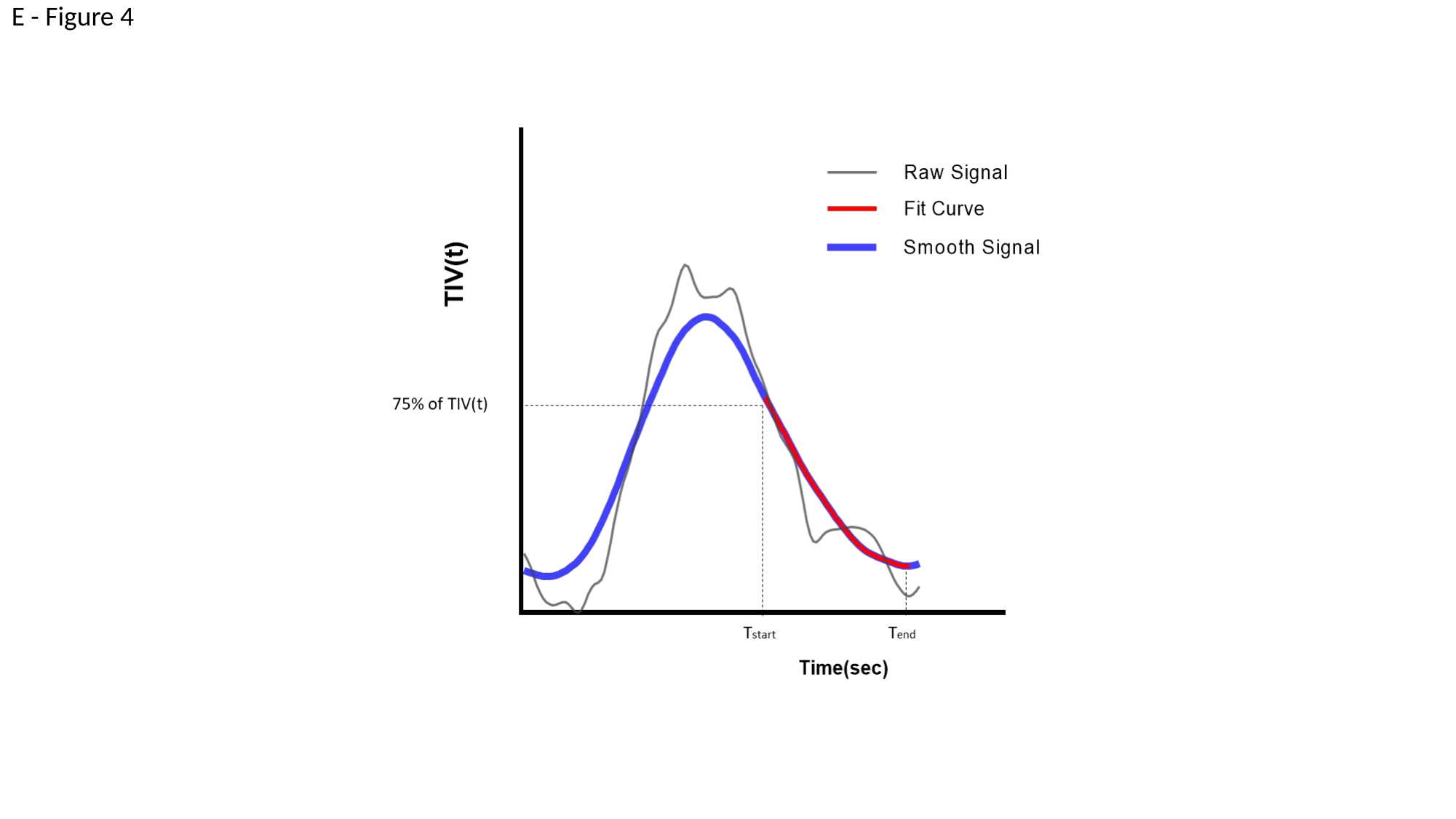

E - Figure 4

## Slide 5
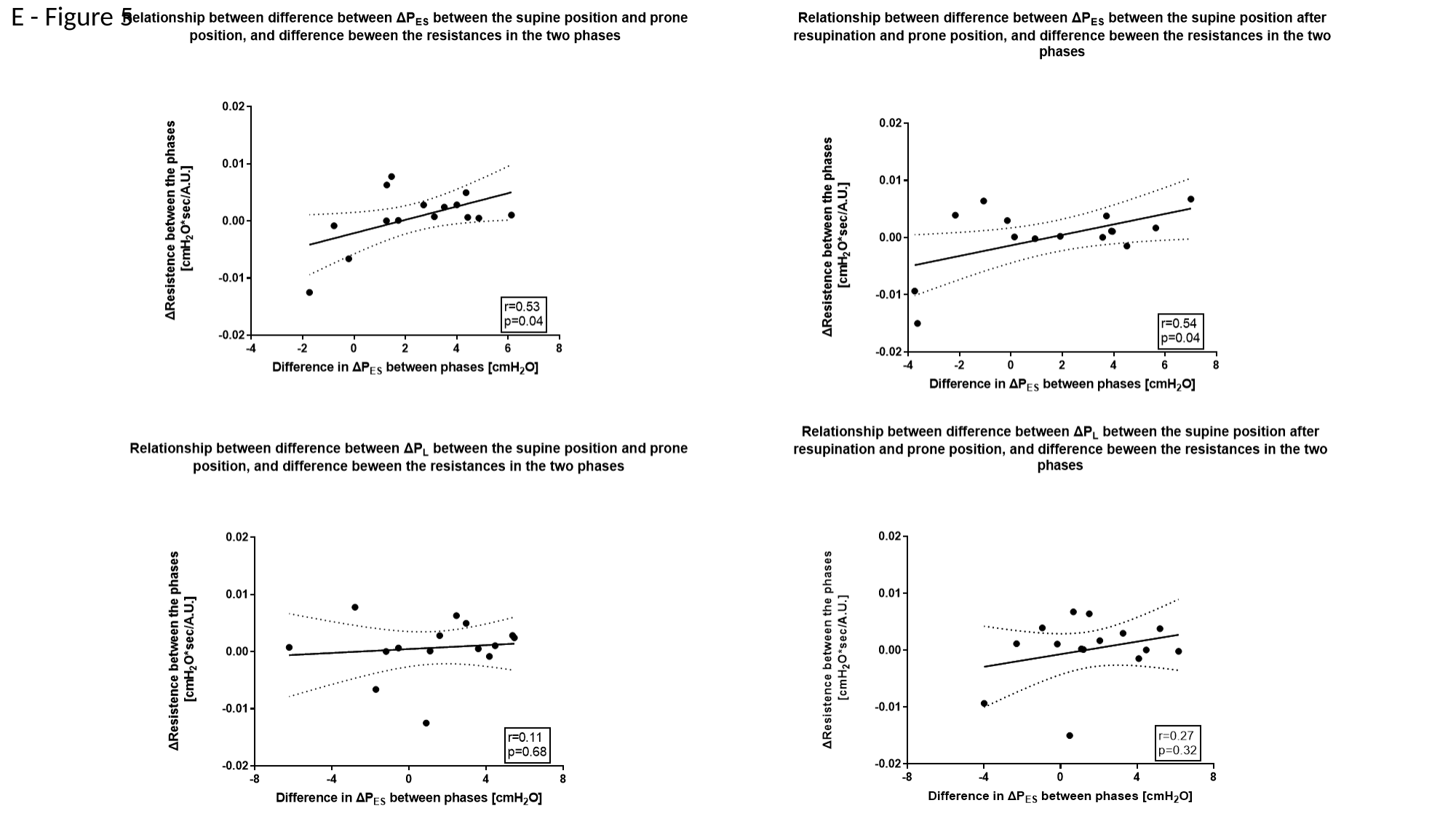

# E - Figure 5

Supplement: Supplementary file 1 — Additional file 1. E – Figure 1: VAS dyspnea scale.E – Figure 2: VAS discomfort scale. E – Figure 3: Graphic representation of the 4 ROIs and lung contour. In the picture there is a representation of the 32 x 32 matrix provided by the FluxMed® device, after filtering for hearth artifacts and isolating the lungs. In order, from ventral to dorsal: ventral ROI, mid-ventral ROI, mid-dorsal ROI, dorsal ROI. In the analysis, as per manufacturer instructions, the EIT value of each pixel was adjusted to its position in the lung image, with the most peripherical and ventral pixels having the lowest value. E – Figure 4: Graphic representation of the exponential fitted curve in a representative single breath cycle of supine position. The thin gray line represents the raw data with artifacts on which a moving-average smoothing is applied (blue line). The red curve is the negative exponential fit applied (eq. 1). The starting point was chosen as the 75% of the maximum value of the TIV and the end point as the minimum value of the TIV(t).E – Figure 5: Relationship between the change in inspiratory effort and the change in resistances across study phases. In the top two panels, a significant linear relationship is depicted between the increase in respiratory system resistances and inspiratory effort when comparing the supine phases (before and after re-supination) to the prone phase. In the bottom two panels, the same relationship is shown between the change in respiratory system resistances and ΔPL2 across the study phases; however, in this case, the relationship is not significant. [file 13054_2023_4600_MOESM1_ESM.pptx]
