# Supplementary material for: Physiological effects of awake prone position in acute hypoxemic respiratory failure
Source: Crit Care. 2023 Aug 17;27:315. doi: 10.1186/s13054-023-04600-9 (PMC10433569; doi:10.1186/s13054-023-04600-9)
Supplement: Supplementary file 2 — Additional file 2. Supplementary material. [file 13054_2023_4600_MOESM2_ESM.docx]

**Supplementary material**

1. **Contraindication to Awake Prone Positioning**

We considered contraindication to awake prone positioning patients that was uncooperative or showed altered mental status, hemodynamic instability, arrythmias and unstable spine, pelvic instability, chest trauma or presence of chest tube, and recent abdominal surgery.

1. **Electrical impedance tomography device and signal sampling**

Electrical impedance tomography (EIT) measurements were performed with the FluxMed® device (MBMED, Buenos Aires, Argentina) which had been calibrated and self-tested according to the manufacturer’s instructions.

The electrode belt was placed as per manufacturer’s instructions around the thorax between the 5th or 6th parasternal intercostal space and connected to a dedicated device to record electrical impedance signals. EIT data were acquired at a frame rate of 40Hz, and with the same spatial resolution and reconstruction algorithms of the other commercially available devices.

All EIT data were saved in real time in the FluxMed® hard drive, downloaded into a personal computer for offline analysis. End-expiratory lung impedance and tidal impedance were recorded continuously during each step, and the last 15 minutes were analyzed.
Mechanical and impedance signals were provided as a 32 x 32 matrix (e.g. 1024 pixels), that was post-processed to decrease the noise excluding non-lung signals (the hearth region and the chest wall) according to the manufacturer’s algorithm, based on anthropometric data. All the signal in the matrix outside the lung contour was equaled to 0. The lung contour did not change in the same patient.
We defined 4 standardized regions in the thorax section: 2 anterior regions (ventral ROI and mid-vental ROI), and 2 posterior regions (mid-dorsal ROI and dorsal ROI). The 4 regions were standardized, and did not change between patients and interfaces, to ensure comparability of EIT data. In dividing the 4 regions we did not follow the classical 8x32 approach, as it can underestimate the ventral and the dorsal ROI. A simplified version of the ROIs distribution is shown in **E-Figure 3**.

The exact number of rows in each ROI were defined as follows, top to bottom **(E-Figure 3)**:

- Ventral: from rows 1-10;
- Mid-ventral: 11-15;
- Mid-dorsal: 16-20;
- Dorsal: 20-32.

After the definition of the lung contour and of the 4 ROIs, the signals were post processed to remove artefacts (e.g. cough, esophageal spasm), to obtain a sequence of at least three minutes for each step of the experimental protocol.

From the tracings, by resampling and interpolating process, we obtained a single “average” breath that we considered highly representative of the patient interaction with each respiratory support interface in the different oxygenation conditions.
With a custom-made MATLAB toolbox, we visually analyzed the EIT and the P_ES_ tracings (the EIT an mechanical signal came out synchronized from the Fluxmed) and manually selected from the last 15 minutes the breaths without clear artifacts (esophageal spasm, cough, etc.).
After the selection, we double checked if P_ES_ and EIT were synchronized, as two different machines may have different clock times, and the connecting cables can introduce an unwanted delay of few milliseconds. We confirmed adequate synchronization visually and evaluated the correlation at the Pearson test, manually adjusting for the few milliseconds of delay when they occurred.
At this point, we defined each breath as a time interval of 1.5 seconds before and after the minimum value on the P_ES_ tracings; we chose the P_ES_ since its signal is usually more stable than the EIT, and given the synchronization of the two traces a breath selected on the P_ES_ trace would reflect the same breath on the EIT trace. The single-breath trace of each breath was visualized, so that we could visually confirm the absence of artifacts, and manually exclude the ones with abnormalities not detected in the first screening (cough, esophageal spasm, patient's movement altering EIT signal, etc).
In case the minimum value on the P_ES_ was misinterpreted by the software and did not correspond to the actual negative peak in P_ES_ during inspiration it was manually set.
Finally, we superimposed the so-selected ‘breaths’ around the minimum value on the P_ES_ trace and obtained the averaged breath for each patient.

EIT measurements were expressed in arbitrary units, the unit commonly used to quantify the amplitude of impedance changes within EIT image pixels.
Current EIT image reconstruction algorithms do not produce images with well-defined units, and their sensitivity can depend on many factors such as subject posture and electrode placement details. It is thus common to express the image units as “arbitrary” (1).

1. **Airway Resistance**

The increase of effort in patient under awake prone position was not accompanied by an increase in transpulmonary pressure. This phenomenon could be explained with the increased airway resistance.

As illustrated in figure **E-figure 4**, we performed a smoothing with a moving average on EIT raw signal on 10 points and obtained smoothed tidal impedance variation (TIV)

We fitted an exponential curve on smoothed EIT-derived signal:

$$\left( \mathrm{eq} 1 \right) {TIV}_{fitted}(t)={\alpha e^{-\frac{t}{\tau}}}$$

where α is the initial value of TIV, and ꞇ is the time constant representing the time for *TIV(t)* to exhale 2/3 of its volume, as done by Guttmann et al. (2).

We obtained the resistance of the airway system in analogy with an RC circuit as follows:

(eq 2) $ꞇ$= R C

where C is the compliance of the respiratory system and R is the flow-dependent airway resistance.

As shown in **E-figure 5**, we found a positive correlation (r=0.53, p=0.04) between the variation of ΔP_ES_ and variation of airway resistance from prone to supine position.

1. **Quantification of Pendelluft %**

Given the high frequency of pendelluft during spontaneous breathing we calculated the breath cycle per each of the pixels provided by the FluxMed® device in the 32 x 32 matrix (**E- Figure 3**).
We defined TidalΔZ as follows:

$$Tidal\Delta Z= \sum_{i=N} Pixel\_{end\_insp Z}_{pixel}- Pixel\_en{d\_expZ}_{pixel}$$

Which is the difference between the impedance value of each pixel at the end of inspiration on the pixel breath cycle – which is pixel’s maximum impedance value - and the impedance value of each pixel at the end of expiration on the pixel breath cycle – which is pixel’s minimum impedance value.
The pendelluft is the difference between the air moving into each region (a single pixel, in our case) and the air moving in the whole lung, as measured by the Global EIT trace, that subtract – per each pixel – the EIT values at the end inspiration and at the end expiration, underestimating the volume moving in the lung if the pixels and the Global EIT trace are off-phased.

Consequently, the pendelluft effect can be calculated as follows:

$$Pendelluft \%=\frac{\sum_{i=N} (Pixel\_{end\_insp Z}_{pixel}- {Pixel\_end\_expZ}_{pixel})- \sum_{i=N} (Global\_en{d\_inspZ}_{pixel} - Global\_{end\_exp Z}_{pixel})}{\sum_{i=N} (Pixel\_{end\_insp Z}_{pixel}- {Pixel\_end\_expZ}_{pixel})} x 100$$

Indeed, in case there is no intra-tidal shift and the lung breath cycle is completely synchronous with each pixel breath cycle, the two methods of calculating TidalΔZ are equivalent and return exactly the same value.

**Supplementary figures legend**

**E – Figure 1:** VAS dyspnea scale.

**E – Figure 2:** VAS discomfort scale.

**E – Figure 3:** Graphic representation of the 4 ROIs and lung contour. In the picture there is a representation of the 32 x 32 matrix provided by the FluxMed® device, after filtering for hearth artifacts and isolating the lungs. In order, from ventral to dorsal: ventral ROI, mid-ventral ROI, mid-dorsal ROI, dorsal ROI.

In the analysis, as per manufacturer instructions, the EIT value of each pixel was adjusted to its position in the lung image, with the most peripherical and ventral pixels having the lowest value.

**E – Figure 4:** Graphic representation of the exponential fitted curve in a representative single breath cycle of supine position. The thin gray line represents the raw data with artifacts on which a moving-average smoothing is applied (blue line). The red curve is the negative exponential fit applied (eq. 1). The starting point was chosen as the 75% of the maximum value of the TIV and the end point as the minimum value of the TIV(t).

**E – Figure 5:** Relationship between the change in inspiratory effort and the change in resistances across study phases. In the top two panels, a significant linear relationship is depicted between the increase in respiratory system resistances and inspiratory effort when comparing the supine phases (before and after re-supination) to the prone phase.
In the bottom two panels, the same relationship is shown between the change in respiratory system resistances and ΔP_L_ across the study phases; however, in this case, the relationship is not significant.

**References**

1. Frerichs I, Amato MBP, Van Kaam AH, Tingay DG, Zhao Z, Grychtol B, *et al.* Chest electrical impedance tomography examination, data analysis, terminology, clinical use and recommendations: Consensus statement of the TRanslational EIT developmeNt stuDy group. *Thorax* 2017;72:83–93.

2. Guttmann J, Eberhard L, Fabry B, Bertschmann W, Zeravik J, Adolph M, *et al.* Time constant/volume relationship of passive expiration in mechanically ventilated ARDS patients. *European Respiratory Journal* 1995;8:114–120.
